# Superconductivity in Bundles of Double-Wall Carbon Nanotubes

**DOI:** 10.1038/srep00625

**Published:** 2012-09-03

**Authors:** Wu Shi, Zhe Wang, Qiucen Zhang, Yuan Zheng, Chao Ieong, Mingquan He, Rolf Lortz, Yuan Cai, Ning Wang, Ting Zhang, Haijing Zhang, Zikang Tang, Ping Sheng, Hiroyuki Muramatsu, Yoong Ahm Kim, Morinobu Endo, Paulo T. Araujo, Mildred S. Dresselhaus

**Affiliations:** 1 Department of Physics and William Mong Institute of Nano Science and Technology, HKUST, Clear Water Bay, Kowloon, Hong Kong, China; 2 Faculty of Engineering and Institute of Carbon Science and Technology, Shinshu University, 4-17-1 Wakasato, Nagano, 380-8553, Japan; 3 Department of Physics and Department of Electrical Engineering and Computer Sciences, Massachusetts Institute of Technology, Cambridge, Massachusetts, USA; 4 W.S. and Z.W. contributed equally to this work.; 5 Current address: Department of Physics, Princeton University, Princeton, NJ, 08540 USA.

## Abstract

We present electrical and thermal specific heat measurements that show superconductivity in double-wall carbon nanotube (DWCNT) bundles. Clear evidence, comprising a resistance drop as a function of temperature, magnetoresistance and differential resistance signature of the supercurrent, suggest an intrinsic superconducting transition below 6.8 K for one particular sample. Additional electrical data not only confirm the existence of superconductivity, but also indicate the *T*_c_ distribution that can arise from the diversity in the diameter and chirality of the DWCNTs. A broad superconducting anomaly is observed in the specific heat of a bulk DWCNT sample, which yields a *T_c_* distribution that correlates well with the range of the distribution obtained from the electrical data. As quasi one dimensionality of the DWCNTs dictates the existence of electronic density of state peaks, confirmation of superconductivity in this material system opens the exciting possibility of tuning the *T*_c_ through the application of a gate voltage.

Double-wall carbon nanotubes (DWCNTs) are the world's smallest concentric cables exhibiting extraordinary thermal stability and mechanical strength^1^,^2^,^3^,^4^,^5^. In terms of their electronic properties, the outer tube may stabilize the metallic inner tube against the Peierls distortion^6^ since the van der Waals or other interactions between the inner and outer tubes tend to detract from the one-dimensionality of the system, which is central to the Peierls mechanism. The outer tube can also offer dielectric screening for the electron-electron interaction^7^ that is in competition with the attractive electron-phonon interaction. Both predispositions are favorable to the prospect of DWCNTs being the world's thinnest superconducting cables. More recently, superconductivity has been predicted in isolated DWCNTs by first-principles calculations^8^. On the negative side, since it is known that the transverse couplings between the nanotubes can be important for the appearance of a resistive superconducting transition^9^,^10^,^11^, therefore unless the outer tube is metallic, such coupling may be reduced. These considerations lead to the conclusion that if superconductivity can indeed exist in DWCNTs, they should appear only in a fraction of the samples. This conclusion also applies to the earlier reports on superconductivity in ropes of single wall carbon nanotubes (SWCNTs)^12^,^13^,^14^ and multi-wall carbon nanotubes (MWCNTs)^15^,^16^, since only ~1/3 of the SWCNTs are expected to be metallic and both the ropes of SWCNTs and MWCNTs arrays have a broad distribution of chiralities and diameters and also display different metallicity. In comparison, the DWCNTs with small diameters (<2 nm) and high purity^1^,^17^ are promising new 1D superconductors.

We have carried out an intensive effort in which ~200 devices of DWCNTs were fabricated for electrical transport measurements, together with thermal specific heat measurements of bulk samples. The results indicate clear electrical transport evidence for superconductivity in about 10% of the samples, and a definitive specific heat anomaly within a temperature range of 4–18 K, in agreement with the range of *T_c_*'s obtained from the electrical data. Our results, first for the DWCNTs, are therefore supportive of the thesis that carbon nanotube arrays can be superconducting^9^,^10^,^11^. Note that pure carbon is not a superconducting element^18^, therefore the superconducting characteristics observed here must arise from nanostructuring.

## Results

### Structural and Raman characterization

**Figure 1:**
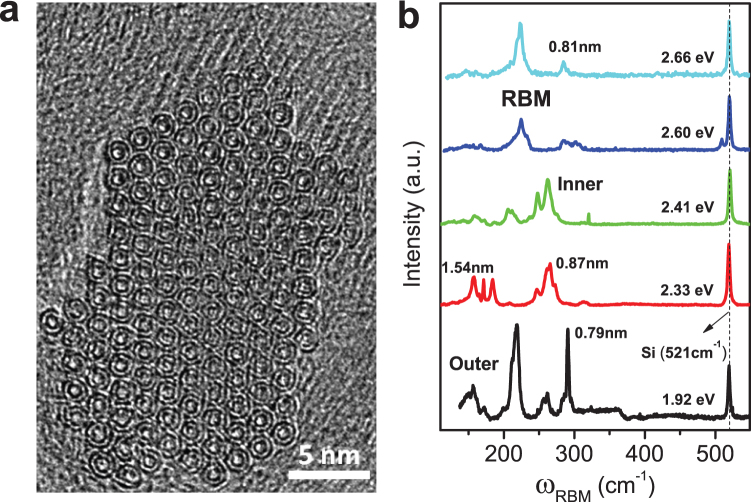
TEM image and Raman spectra of DWCNTs. (a) Cross-sectional HRTEM image of a single bundle, showing the nearly perfect arrangement of about 150 DWCNTs with similar diameters. (b) Resonant Raman spectra, using different laser excitation energies, of a bulk sheet of DWCNTs, indicating different diameters and chirality combinations of the inner and outer tubes.

The DWCNTs were fabricated using an optimized catalytic chemical vapor deposition (CVD) method and the nanotubes were then processed through a highly efficient purification process^19^,^20^. Figure 1a shows a cross-sectional high-resolution transmission electron microscope (HRTEM) image of a DWCNTs bundle. It indicates the perfect close-packed arrangement of the DWCNTs, forming a nearly crystal-like structure which also has been confirmed by X-ray diffraction^4^. The diameters of the outer and inner tubes were measured from the HRTEM image to be around 1.54 nm and 0.83 nm, respectively. Figure 1b shows the low frequency region of the resonant Raman data for a bulk sheet of interwoven DWCNTs. The tube diameter 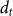 [nm] can be calculated using the equation 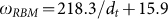, where 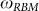 is the radial breathing mode (RBM) frequency [cm^−1^]^21^. The higher frequency RBM is associated with the inner tube, while the lower frequency is related with the outer tube. It is seen that several kinds of DWCNTs with different diameters and chirality combinations of inner and outer tubes exist in the bulk sheet. The higher frequency region of the resonant Raman data, not shown here, exhibits negligible D band intensity compared with that of the G band, showing the high quality of the DWCNTs. A more detailed structure characterization will be presented separately in another manuscript.

### Electrical characteristics

We present measured results on five devices made from single DWCNT bundles by an *e*-beam lithography and lift-off process. The device characteristics are summarized in Table 1, with the diameter of the bundles measured by atomic force microscope (AFM) in each case.

**Figure 2:**
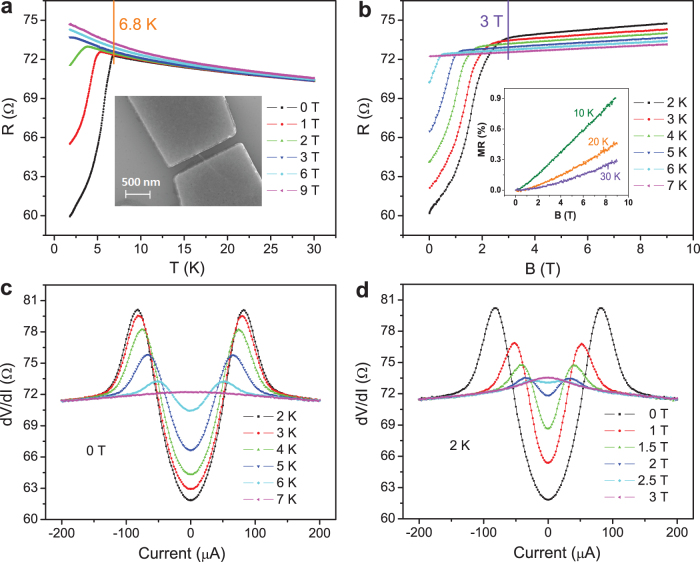
Electrical transport data of a single DWCNT bundle sample Z-14. (a) Resistance as a function of temperature at a magnetic field B = 0, 1, 2, 3, 6, and 9 T, (left) from bottom to top, measured with a 1µA DC current. The inset shows its SEM image. (b) Magnetoresistances at temperature T = 2, 3, 4, 5, 6, and 7 K, (left) from bottom to top. The inset shows magnetoresistances at a higher temperature range T = 10, 20 and 30 K, from top to bottom. (c) Differential resistance at zero magnetic field as a function of current at temperature T = 2, 3, 4, 5, 6 and 7 K, (center) from bottom to top. (d) Differential resistance at 2 K as a function of current at a magnetic field B = 0, 1, 1.5, 2, 2.5, and 3 T, (center) from bottom to top.

Figure 2 shows the electrical transport data measured from a two-probe device Z-14. The inset to Figure 2a shows the scanning electron microscope (SEM) image of the device after measurement, where the single bundle is clearly seen to be between two electrodes with a separation of 240 nm. The device was annealed under an ultra-high vacuum (see Methods section), which greatly enhanced the contacts between the nanotube bundle and the electrodes. This sample has been characterized by Raman spectra. Details are given in the Supplementary Information. The Raman results indicate the inner tube to be metallic, but the character of the outer tube can be either metallic or semiconducting. However, since the outer tubes used (in growing the inner tubes) for this DWCNT batch is the high-purity metallic carbon nanotube (98%, NanoIntegris Corp.), it is most likely that the outer tubes of Z-14 are also metallic in character.

Figure 2a shows the temperature dependence of the resistance measured with a DC current of 1 µA. A sharp drop is seen to initiate at ~6.8 K, and the transition was observed to move to lower temperatures when a magnetic field was applied perpendicular to the nanotube axis (the default field direction in this article). At zero magnetic field, the resistance drops ~18% from 6.8 K to 2 K and does not show signs of leveling off. With a magnetic field larger than 3 T, the resistance drop is largely suppressed above 2 K. By approximating bundle's cross-sectional shape as circular with a diameter of 15 nm, we estimate the number of DWCNTs to be ~60. From the measured normal state resistance of 72 Ω, each DWCNT should have a resistance of 4.2 kΩ. This value is only slightly larger than the 3.3 kΩ for the minimum ballistic quantum resistance, as each DWCNT has four conducting channels. Our estimate thus implies that the mean free path in Z-14 is fairly large—on the order of 100–200 nm. Figure 2b shows the magnetoresistances (MR) at different temperatures. At 2 K, there is a clear turning point at 3 T but the MR still remains positive above that. The inset shows the MR at a higher temperature range from 10 K to 30 K. We can see a small positive magnetic field dependence of the resistance even at 30 K. Extrapolating the turning point field at each temperature to 0 K, we obtain the critical magnetic field to be 4.4 Tesla. By following the calculation of the parallel critical field of a thin film^22^, the perpendicular critical field for a thin wire is given by 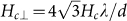, where *H_c_* is the thermodynamic critical field, 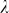 is the magnetic penetration length, and *d* is the diameter of the thin bundle. Since the coherence length of the wire is given by 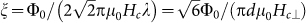, where 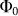 is the quantum flux, *d* = 15 nm and 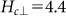 Tesla, we get its value to be 24.3 nm. This is noted to be on the same order of magnitude as the superconducting coherence length in 4-Angstrom SWNTs (6–15 nm)^11^,^23^ and B-doped SWNTs (17 nm)^24^. From this value of the coherence length we may also estimate the inverse proximity effect, since for our sample normal metal electrodes were used, and the critical temperature *T_c_* of a superconducting wire would consequently be suppressed in accordance with the formula 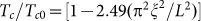
25, with *T_c0_* being the critical temperature in the absence of the inverse proximity effect. From the value of the coherence length and *L* = 240 nm, we obtain *T_c_* = 0.75 *T_c0_* in our case.

Figure 2c shows the differential resistance at zero magnetic field as a function of the bias current for different temperatures. A resistance dip centered at zero bias current appears at 2 K and gradually vanishes as the temperature increases to 7 K, in agreement with the temperature dependence of the resistance. This dip in differential resistance is usually taken as a sign of the appearance of a supercurrent.

In Fig. 2c the critical current is seen to be 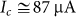 at 2 K, which translates to a critical current density 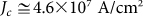. It is known that the critical current density is much smaller than the depairing current density in bulk samples, owing to the movements of vortices as well as the non-uniformity of the current. However, it has been demonstrated that the depairing current density can indeed be reached in narrow strips of superconducting thin films, but not in the wide strips^26^,^27^. Since our DWCNT bundle may be regarded as the limiting case of a narrow strip of superconducting thin film, it is thus reasonable to treat the measured critical current in our ultra-thin DWCNTs bundle as the depairing current. From the expression 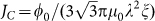
28, we can deduce the magnetic penetration depth 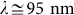. Hence the Ginzburg-Landau parameter 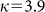, i.e., the DWCNTs in this case belong to type-II superconductor.

Figure 2d shows the differential resistances at 2 K as a function of the bias current under different magnetic fields. The resistance dip gradually becomes less pronounced and disappears completely as the magnetic field increases to 3 T, in agreement with the transition point in Fig. 2b. Above 3 T, a resistance peak arises. This type of differential resistance peak behavior was also observed in the earlier reports^11^,^12^,^15^ on nanotube superconductivity.

Besides the device Z-14, below we also present the results on 4 other devices, out of 22 samples that display superconducting signals. Most of these data have less quality than Z-14, but they still show a clear evidence of superconductivity.

**Figure 3:**
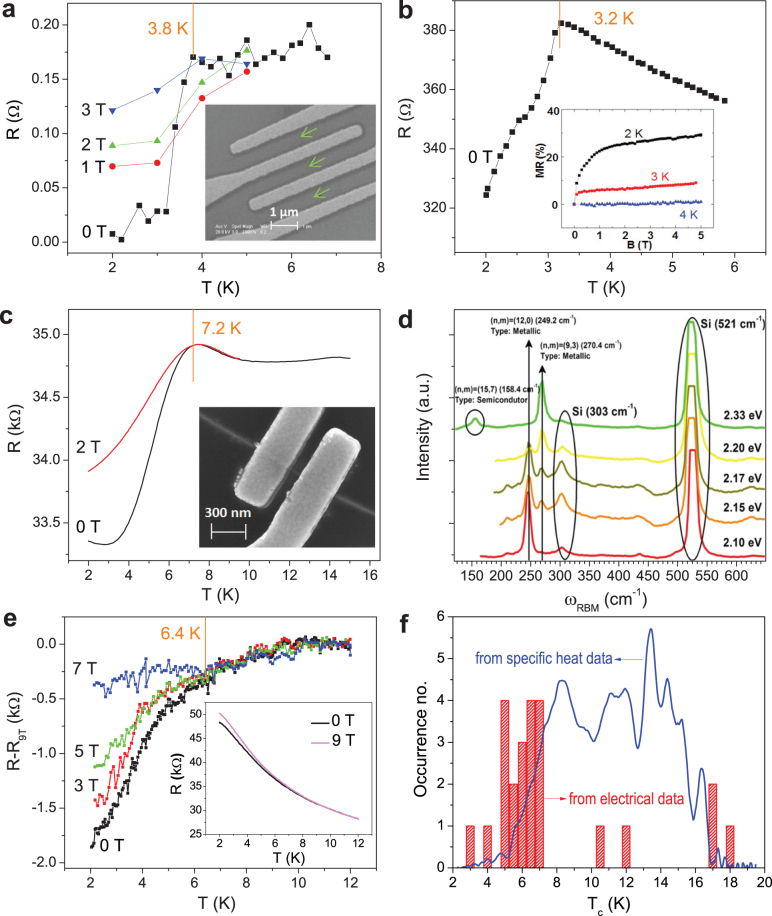
Resistance vs. temperature curves, resonant Raman spectra, and histogram of transition temperatures. (a) Four-probe resistance for sample DW-1 as a function of temperature at a magnetic field B = 0, 1 , 2, and 3 T, (left) from bottom to top, measured with a 5 µA DC current. The inset shows its SEM image. (b) Two-probe resistance for sample DW-2 as a function of temperature at zero field, measured with a 1µA DC current. The inset shows the MR measured at different temperatures, defined as (R_B_−R_0_)/R_0_×100%. (c) Two-probe resistance for sample G-5 as a function of temperature at B = 0, 2 T, measured by applying a 10 nA AC current. The inset shows its SEM image. (d) Resonant Raman characterization for the same sample G-5, showing (12,0) and (9,3) as the possible inner tubes, while the outer tube can be (15,7), which is semiconducting. (e) Two-probe resistance for sample Y-2 as a function of temperature, measured with a 100 nA DC current. Black, red, green and blue lines represent, respectively, the difference of resistance at B = 0, 3, 5 and 7 T for data taken at B = 9 T. The inset shows the original resistance vs temperature curves at B = 0 and 9 T. The short vertical lines in (a), (b), (c) and (e) indicate the transition temperatures *T_c_*. (f) Histogram of transition temperatures observed in 22 samples. Blue curve is the calculated *T_c_* distribution dF/dT from the specific heat data (see Fig. 4c). While the range of the *T_c_* distribution is the same in the two cases, it can be seen that the electrical measurements have missed a large fraction of the superconducting samples. The reason is speculated to be that the “missed” samples may have large phase slip-induced resistances, owing to large 1D fluctuations. Since specific heat is much less sensitive to phase fluctuations, such measurement would be able to capture most of the superconducting DWCNTs.

Figure 3a,b shows the temperature dependence of resistance for devices DW-1 and DW-2, respectively. The inset to Fig. 3a shows a SEM image of the four-probe device DW-1, made with a 500 nm separation between two inner electrodes. At zero field, the resistance decreases drastically below 3.8 K for DW-1 by more than 1 order of magnitude to nearly zero, while the resistance of DW-2 displays a sharp drop initiating at 3.2 K. The positive MR below the transition temperatures is another indication of superconductivity for both DW-1 and DW-2, while above the transition temperature the MR is nearly zero.

All three samples presented above have a small resistance, less than 1 kΩ at room temperature. In particular, DW-1 displayed ballistic conductance under four probe measurements with a fraction of an Ohm. It exhibits a sharp, order of magnitude drop to near-zero resistance at 3.8 K. Moreover, the small resistance means that both the outer and inner tubes must be metallic, and in this case the sharpness of the transition is in support of the possibility of true 1D superconductivity in a single DWCNT or a few DWCNT. DW-1 is the only sample, among all the samples fabricated and tested, that displayed ballistic behavior of very small resistance. For DW-2, the bundle is longer in length and the transition is more sensitive to magnetic field. The low resistances for both Z-14 and DW-2 at low temperatures also imply that the measured DWCNTs may have metallic outer tubes.

However, in general, most of the other samples have room temperature resistances larger than several kΩ either in the four-probe or the two-probe configuration. For these samples it is difficult to observe a sharp drop or even a direct decrease in resistance as a function of temperature, even if the inner tubes are in the superconducting state, as the outer tubes are likely to be semiconducting. Figure 3c shows the resistance of another sample G-5, made with a 100 nm separation between two contacts as shown in the inset. A repeatable finite resistance drop below 7.2 K was observed in this sample, and the resonant Raman spectra of the bundle are shown in Figure 3d. The inner tube can be either (12,0) or (9,3), both metallic with diameters of 0.95 nm and 0.86 nm, respectively, which is possibly the origin of superconductivity, while the outer tube is (15,7) with a diameter of 1.54 nm, which is semiconducting. (The tube diameter 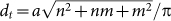 [nm], where 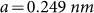 is the lattice constant^29^) Given that the inner and outer nanotubes should differ in diameter by at least 0.68 nm, (9,3) is a more likely candidate for the inner tube. While we can see a smooth resistance drop in the case like sample G-5, many other samples did not show a direct resistance decrease with temperature down to 2 K. Nevertheless, they exhibit positive MR behavior like the sample device Y-2 shown in Figure 3e. By subtracting the measured resistance at 9 T (at which field the superconductivity should be considerably suppressed, thus it is treated approximately as the normal state background), it is seen that below 6.4 K the resistance increases as the magnetic field is increased. This temperature-dependent MR feature, which vanishes above a transition temperature, also offers support to the existence of superconductivity in DWCNTs.

**Figure 4:**
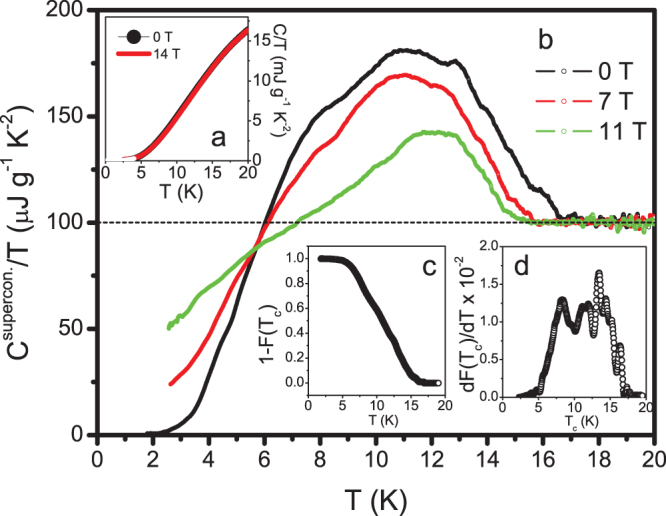
Specific heat data. (a) Total specific heat of our macroscopic sample of DWCNTs in a field of 0 and 14 T. The white line represents a fit according to 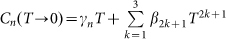. (b) Electronic specific heat derived from the difference between data in 0 and 14 T, 7 and 14 T as well as 11 and 14 T and the γ_n_ value of the fit in (a). (c) Temperature dependence of the superconducting fraction 1-*F*(*T*) in our macroscopic sample as obtained from direct deconvolution of the specific-heat data (see text for details). (d) The corresponding *T*_c_ distribution d*F*/d*T*.

Figure 3f is a histogram of the transition temperatures observed in all the samples that exhibit evidence of superconductivity. It is seen that the transitions occur in a wide temperature range. Such diversity of transition temperatures is expected, owing to the different inner tube diameters/chiralities that can have a direct bearing on the superconducting transition^30^. The blue curve in Fig. 3f is the *T*_c_ values deduced from the specific heat signal (see Fig. 4c), which spreads over the same wide temperature range, although it shows far more weight in the higher temperature range. While the range of the distribution is the same in the two cases, the electrical measurements apparently missed many superconducting DWCNTs. The reason for this difference between the two distributions is discussed below.

For the other ~180 samples that did not exhibit superconducting characteristics, the resistive behavior is uniformly insulating in character, due either to semiconducting DWCNTs or the blocking contacts. Some devices can reach resistances on the order of megaohm or more at low temperatures.

### Thermal specific heat characteristics

A definitive probe of superconductivity typically requires the observation of zero-resistivity, the observation of the magnetic Meissner effect and the observation of the specific-heat anomaly. While the first two probes may be cheated in bulk samples by percolative or surface superconductivity^31^, the specific heat (which averages over the whole volume of the sample) is generally regarded as a true bulk probe of superconductivity. In the present case, measurements of the magnetic Meissner effect are hampered by the strong background contribution of spurious magnetic nanoclusters which were used as catalysts in the sample fabrication process. Therefore, the investigation of the specific heat is of particular importance. To probe the specific-heat transition in bundles of DWCNTs represents, however, a particular challenge due to the expected small size of the signal: A macroscopic bulk sample will certainly contain a mixture of different DWCNTs of different chiralities and only a fraction of them (~10% according to our resistivity result) may become superconducting. In order to overcome this difficulty, we used our home-made ultra-high resolution AC specific-heat technique^32^ combined with a highly accurate ‘long' relaxation technique^33^,^34^ on a macroscopic sample of 5 mg DWCNT bundles.

The observation of such a tiny superconducting anomaly requires the subtraction of normal state reference data taken in a sufficiently high magnetic field to quench superconductivity. Our maximum field was limited to 14 T which, according to our resistivity data, is expected to be sufficiently high to restore the normal metallic state. The measurement required careful calibration of the field dependence of our thermometer, which was done with the help of an Ag calibration sample in a separate measurement.

Figure 4a shows the total heat capacity in 0 and 14 T. A small difference (1.5% at 10 K) is found, which can however be clearly resolved with our high-resolution AC specific-heat technique. If we assume that the applied magnetic field of 14 T is sufficient to fully suppress superconductivity, we can analyze the normal-state specific heat in a standard way according to the expansion: 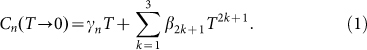
The first term is the electronic Sommerfeld contribution with 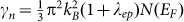, *k_B_* is Boltzmann's constant, *λ_ep_* is the electron-phonon coupling constant, and *N*(*E_F_*) is the electronic density of states at the Fermi level including the two spin directions. The second term in Eq. (1) is the low-temperature expansion of the lattice specific heat, where 
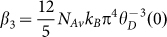
, with *N_Av_* being Avogadro's number and *θ_D_*(0) is the initial Debye temperature. From a fit from 2 K to 4 K of the 14 T data shown in Fig. 4a, we obtain a total γ_n_ = 140 ± 40 μJ/g K^2^. The relatively large error is due to the tiny value of γ_n_ which reaches the limit of our precision.

The main Fig. 4b shows the electronic specific heat *C*^electr.^/*T* in zero magnetic field, 7 T and 11 T derived by subtracting data in a field of 14 T as normal state background, and adding a constant (100 μJ/g K^2^) that is the difference between the low and high temperature values of the specific heat anomaly, which is the change in the Sommerfeld constant induced by superconductivity. A very broad superconducting transition is observed with onset at ~17 K. The total change in the Sommerfeld constant, 100 μJ/g K^2^, when compared to the total γ_n_ of 140 μJ/g K^2^, suggests that a large fraction of all the metallic nanotubes in the sample becomes superconducting at low temperatures. Since only ~10% of the samples exhibit an electronic transport manifestation of superconductivity as mentioned previously, we conclude that only slightly greater than one tenth of the superconducting DWCNTs were accessed via transport measurements. It is speculated that much of the “missed” superconducting bundles could be exhibiting 1D fluctuating superconductivity in which the samples display finite resistance owing to phase slips^22^. Since the superconducting specific heat anomaly is much less sensitive to phase fluctuations, such measurement can naturally capture most, if not all, of those samples.

Owing to the broadening, it is on first view not clear if the specific heat satisfies the consistency relationship between the magnitude of the signal and γ_n_ as in the case of BCS theory (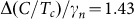). We will show later that the reason for the broad transition is a broad *T*_c_ distribution. Therefore, the relation would need to be tested for each contribution with different *T*_c_ separately. At low temperatures this specific heat component increases approximately exponentially indicating a node-less s-wave nature of the order parameter. Application of a magnetic field has only a minor effect on the onset temperature of superconductivity but shifts the relative weight of the transition anomaly towards lower temperatures. This is mainly a consequence of the random orientation of the DWCNTs in our macroscopic sample which, as quasi-one-dimensional objects, certainly exhibit strongly anisotropic critical fields. Furthermore, contributions from DWCNTs with lower *T*_c_ may have lower critical fields so that the effect of an applied magnetic field shows up stronger at lower temperatures.

The overall shape of the zero-magnetic field anomaly resembles a strongly broadened BCS curve with several distinct shoulders or bump-like anomalies which cannot be described by a *single* BCS curve. The most likely explanation for the shoulders is a broad *T*_c_ distribution. Therefore, we attempt a method which allows us to derive the *T*_c_ distribution directly from our specific-heat data based on the method introduced by Y. Wang et al.^35^ that has been successful in deconvolving the *T*_c_ distribution in a variety of superconducting samples. In the following, we would like to recall this method in order to apply it to our data. In analogy to Ref. 35 we assume that in our macroscopic sample the distribution of *T*_c_ values in different bundles of DWCNTs ranges from 0 to a maximum *T*_c,max_ with partial weights *f*(*T*_c_). The electronic specific heat *C*_e_(*T*) can then be written as: 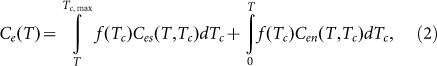
with *C*_es_(*T*,*T*_c_) and *C*_en_(*T*,*T*_c_) the specific heats of electrons in the superconducting state and the normal state, respectively. In a similar way the entropy *S*_e_(T) is given as: 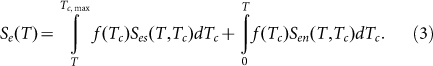
Here *S*_es_(*T*,*T*_c_) and *S*_en_(*T*,*T*_c_) are the entropies of electrons in the superconducting and the normal state, respectively. For the analysis of our sample we need to assume that the normal-state electronic specific-heat coefficient γ does not vary too much among the different bundles, so that approximately: *C_en_*(*T*,*T*_c_) = *C_en_*(*T*) = γ*T*. Furthermore, we assume that the electronic specific heat in the superconducting state can be described by a generalized two-fluid model: 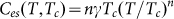. With this expression, the integral *F*(*T*) of the *T*_c_ distribution from 0 to *T* is obtained in the following way: 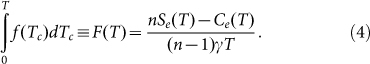
Here *F*(*T*) represents the fraction of our macroscopic sample with *T*_c_ smaller than *T*. The parameter *n* is chosen here as 3.7 in order to normalize the function: 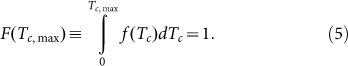
It should be noted that for this normalization and the following analysis we only consider the volume fraction of the sample that becomes superconducting at finite temperatures. We do not consider the large fraction of material that does not become superconducting at all. In Figure 4c we present 1−*F*(*T*) which represents the temperature dependence of the superconducting fraction in the sample and d*F*/d*T* in Figure 4d, which can be directly interpreted as the *T_c_* distribution.

## Discussion

The calculated distribution d*F*/d*T* from the specific heat data is furthermore shown in Fig. 3f for comparison with the statistics of our electrical data. The total width of this *T*_c_ distribution is in good agreement with the *T*_c_ distribution extracted from the resistivity data we collected from many different bundles. However, the distribution obtained from the specific-heat data has more weight in the temperature range of 8-17 K. This could mean that we missed bundles of higher *T*_c_'s in our resistance measurements for either experimental or physical reasons, e.g., owing to phase slip-induced resistance in those samples that exhibit large 1D fluctuations as mentioned earlier.

In view of the above electrical and thermal specific heat evidence, we conclude that a DWCNT can indeed be superconducting, even though its intrinsic mechanism still needs to be further investigated. In particular, the broad distribution in the observed transition temperature raises the possibility that it might be affected by the interaction between the inner and outer tubes. Superconducting DWCNTs open up both basic scientific and potential applications prospects. For the former, doping effects constitute an obvious topic, with the possibility of altering or even enhancing the *T*_c_. For the latter, provided that the contact problem can be resolved and also the superconducting species can be predominantly fabricated, one can envision the potential use of superconducting DWCNTs as the interconnects in nanoelectronic devices.

## Methods

### Sample preparation

We have prepared highly pure (ca. 97%), highly crystalline DWCNTs (absence of the D-band in their Raman spectra) by catalytic chemical vapor deposition and by following the oxidative purification process^1^, in which nanotubes with an outer diameter of ca. 1.6 nm were packed in hexagonal arrays within the bundles. The DWCNTs used in Z-14 sample were prepared by thermally treating high-purity metallic SWCNTs (98%, NanoIntegris Corp.) encapsulating fullerenes at 1500°C, as described in reference 17. After thermal treatment, the growth of DWCNTs from the coalescence of fullerenes in the hollow core of SWCNTs was confirmed by TEM and Raman measurements.

### Device fabrication and transport measurements

To fabricate the sample for transport measurements, we used a small amount of the nanotubes which were dispersed in a dichloroethane solution. After several cycles of sonication (each cycle with 30 minutes sonication and 10 minutes pause), we deposited the solution onto prepared silicon wafers covered with a thermal oxide layer. In this step, we used a spin-coater to make sure that the nanotubes were randomly distributed. Then we used AFM to identify a single DWCNT bundle with 5~10 μm length. Subsequently, Cr/Au (5 nm/40 nm) contacts were made using a standard *e*-beam lithography and lift-off process. All the samples were then annealed in a vacuum of 10^−6^ Torr at 500°C for 1 hour. Exceptionally, device Z-14 was recently made and annealed in an ultra-high vacuum of 10^−9^ Torr (using ion pump) at 600°C for 3 hours. And Ti/Pt (10 nm/ 60 nm) contacts were used for Z-14. All the transport measurements were carried out in a Quantum Design Physical Property Measurement System (with a lowest temperature of 1.8 K and a highest magnetic field of 9 T) with an external Keithley 2182A nanovoltmeter, a 6221 AC/DC current source, and a Stanford Research Systems SR850 lock-in amplifier.

### Specific-heat measurements

To probe the tiny specific-heat anomaly in bundles of DWCNTs with both high accuracy and ultra-high sensitivity (signal-to-noise ratio), we combined two home-made calorimetric techniques with which we have long experience on numerous superconductors in our dedicated specific-heat laboratory (see e.g. Refs. 10, 33, 34). The ultra-high sensitivity for resolving relative changes in the specific heat with respect to a change in temperature or applied magnetic field is provided by an AC modulated-temperature technique^32^, which has been proven to be useful earlier on measuring the specific-heat anomaly of superconducting 4-Angstrom carbon nanotubes embedded in AlPO_4_-5 zeolite crystals^10^. As the AC technique is less accurate in determining absolute values, hence we have combined it with our ‘long' relaxation technique^33^,^34^ which provides the accurate absolute values. The AC calorimeter is constructed from a sapphire chip suspended on 4 phosphor-bronze wires with a resistive heater on the backside. A chromel–AuFe0.07% thermocouple is used to measure the relative temperature difference between the chip and a thermal bath. To achieve the high resolution, a Model A20 DC Nanovolt amplifier from EM Electronics (EM The Rise, Brockenhurst, Hampshire, SO427SJ, England) is used to pre-amplify the small thermocouple voltage. These amplifiers are free from the 1/*f* noise, but their frequency and phase response needs to be, and have been, calibrated in the vicinity of their cutoff frequency (~1 Hz).

## Author Contributions

W Shi is responsible for most of the device fabrication and their measurements, Z Wang is responsible for the fabrication and measurements of Z-14, shown in Fig. 2. Q Zhang is responsible for the data shown in Fig. 3(a) and 3(b). Y Zheng is responsible for samples fabrications and electrical measurements. R Lortz, C Ieong and M He are responsible for the specific heat measurements, Y Cai and N Wang are responsible for the TEM characterization (Fig. 1(a)), T Zhang, H Zhang and Z Tang are responsible for the Raman characterization (Fig. 1(b)), H Muramatsu, Y A Kim and M Endo are responsible for all the DWCNT samples, P T Araujo and M S Dresselhaus are responsible for the Raman characterization of Z-14 and G-5. P Sheng is responsible for initiating and designing the research, and for writing and integrating the manuscript. The following have contributed to writing parts of the manuscript and its multiple revisions: W Shi, Z Wang, Q Zhang, Y Cai, N Wang, R Lortz, P Sheng, H Muramatsu, Y A Kim, P T Araujo, and M S Dresselhaus.

## Supplementary Material

Supplementary Information Superconductivity in Bundles of Double-Wall Carbon Nanotubes

## Figures and Tables

**Table 1:** Device characteristics for single DWCNT bundles used in this article

Sample devices	Number of leads	Materials of leads	Diameter (nm)	Electrode separation (μm)
Z-14	2	Ti/Pt	~15	0.24
DW-1	4	Cr/Au	~5	0.5
DW-2	2	Cr/Au	~10	2.3
G-5	2	Cr/Au	~10	0.1
Y-2	2	Cr/Au	~3	0.2
