# Supplementary material for: Superconductivity in Bundles of Double-Wall Carbon Nanotubes
Source: Sci Rep. 2012 Sep 3;2:625. doi: 10.1038/srep00625 (PMC3432458; doi:10.1038/srep00625)
Supplement: Supplementary Information — Superconductivity in Bundles of Double-Wall Carbon Nanotubes [file srep00625-s1.doc]

**Supplementary Information for**

**Superconductivity in Bundles of Double-Wall Carbon Nanotubes**

Wu Shi^1^, Zhe Wang^1^, Qiucen Zhang^1,2^, Yuan Zheng^1^, Chao Ieong^1^, Mingquan He^1^, Rolf Lortz^1^, Yuan Cai^1^, Ning Wang^1^, Ting Zhang^1^, Haijing Zhang^1^, Zikang Tang^1^, Ping Sheng^1^,* , Hiroyuki Muramatsu^3^, Yoong Ahm Kim^3^, Morinobu Endo^3^, Paulo T. Araujo^4^, and Mildred S. Dresselhaus^4^

^1^*Department of Physics and William Mong Institute of Nano Science and Technology, HKUST, Clear Water Bay, Kowloon, Hong Kong, China*

^2^*Present address: Department of Physics, Princeton University, Princeton, NJ, 08540 USA*

^3^*Faculty of Engineering and Institute of Carbon Science and Technology, Shinshu University, 4-17-1 Wakasato, Nagano, 380-8553, Japan*

^4^*Department of Physics and Department of Electrical Engineering and Computer Sciences, Massachusetts Institute of Technology, Cambridge, Massachusetts, USA*

^*^email: [sheng@ust.hk](mailto:sheng@ust.hk)

**Raman Characterization of Z-14.** To characterize the chirality of device Z-14, nine different laser lines (488nm, 570nm, 579nm, 750nm, 760nm, 770nm, 780nm and 790nm) were used to study its Raman features. Only the laser line 532nm (2.33eV) was found to be in resonance with the tubes, and the corresponding data are shown in Figure S2. Because the laser spot (1µm) is larger than the separation between the two electrodes (240 nm), when the laser power was increased beyond 0.8 mW, the electrodes melted. With lower laser power, we can not observe the RBM signal on this particular device.

However, we do have the G band signal on Z-14, and some limited conclusion can be drawn from that data. The following analysis is based on reference [PRB 82, 155416(2010)], where the isolated DWNTs bundles are studied using different laser lines. Because the G’-band feature has a very specific relation with tube diameter$\omega_{G^{'}}=2700.1-35.4/d_{t}$, its analysis becomes important to shed light on the possible DWNTs configurations as well as their metallicity. It is worth commenting that while this analysis does not provide the right (n, m) indices for the tubes, it gives, with good accuracy, the metallicity of the tubes present on the sample. Based on the relation $\omega_{G^{'}}=2700.1-35.4/d_{t}$ , the possible d_t_ ranges for inner/outer tubes is listed in Supplementary Table S1. After determining the possible range of diameter for tubes constituting the sample, the Kataura's plot is used as a tool to figure out the possible configurations and metallicities of the inner and outer tubes, as shown in Supplementary Fig. S1. The laser line 532 nm is represented by the green line, while the wine and yellow lines gives the limits for a resonant G and G' processes to occur. The dashed rectangles give the possible inner and outer tubes. We can see that the inner tubes are most likely metallic while the outer tubes can be either semiconducting or metallic. It is noted that the dashed rectangles are above the laser energy line because we are dealing with a scattered resonance in a Raman Stokes process. We know that the resonance is with the scattered line because of the absence of the RBM feature, and in a Stokes process the scattered light has the energy increased by the phonon energy (Phys. Reports 409, 47 (2009)).

**Supplementary Table S1.** Possible diameter of inner/outer tubes in sample Z-14.

| **G’(cm^-1^)** | 2642.42 | 2657.08 | 2680.53 |
| --- | --- | --- | --- |
| **d_t_(nm)** | 0.85 | 0.89 | 1.80 |


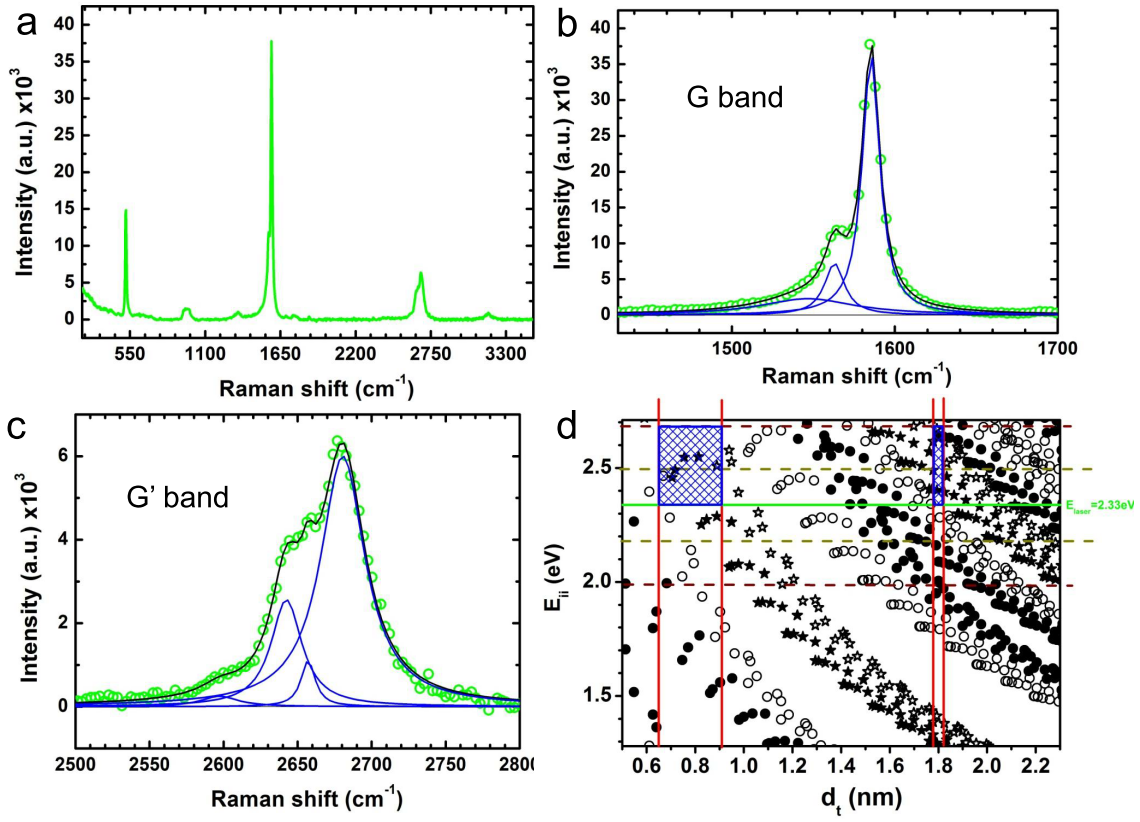


**Supplementary Figure S1*.* Raman characterization of sample Z-14.** (**a**) full range, (**b**) G band range, (**c**) G’ band range and (**d**) Kataura’s plot. The used laser line is 532 nm and the power is 0.8 mW. In (**d**), the stars denote the metallic nanotubes and the circles denote the semiconducting ones. The green line represents the laser line.
